# Superstructure Formation through Coupled Anion and Cation Ordering in Cu-Substituted Lead Oxyapatites

**DOI:** 10.1021/acs.chemmater.4c03130

**Published:** 2025-04-23

**Authors:** Jan P. Scheifers, Adam J. D. Richardson, Hai Lin, Hongjun Niu, Luke M. Daniels, Michael W. Gaultois, Jonathan Alaria, Craig M. Robertson, John B. Claridge, Matthew J. Rosseinsky

**Affiliations:** a Leverhulme Research Centre for Functional Materials Design, University of Liverpool, Materials Innovation Factory, Liverpool L69 7ZD, United Kingdom; b Department of Chemistry, University of Liverpool, Crown Street, Liverpool L69 7ZD, United Kingdom; c Department of Physics, University of Liverpool, Oliver Lodge, Oxford Street, Liverpool L69 7ZE, United Kingdom

## Abstract

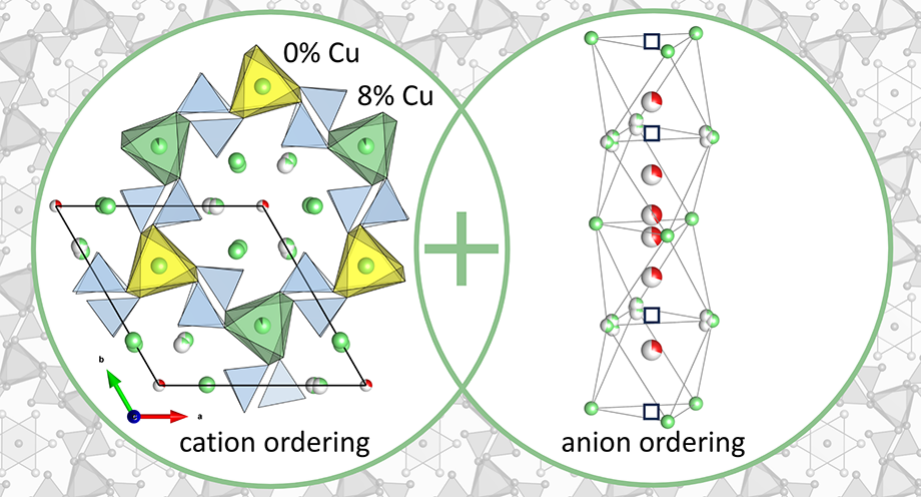

Apatites are an important mineral-based material family with huge chemical and structural diversity. They were recently implicated in the claims of high-temperature superconductivity in materials labeled LK-99 that display complex phase mixtures containing Pb, Cu, phosphate, and oxide components. We report Cu-substituted lead apatite solid solutions Pb_10–*x*_Cu_*x*_(PO_4_)_6_O that display two distinct compositional ranges differentiated by structural ordering. For *x* > 0.5, we observe substitution in the apatite archetype structure, whereas for *x* < 0.5, we find an apatite superstructure with coupled anion and cation ordering. The 1 × 1 × 2 superstructure in the noncentrosymmetric space group *P*6̅ (no. 174) for Pb_10–*x*_Cu_*x*_(PO_4_)_6_O with *x* < 0.5 exhibits a unique oxygen ordering motif in the hexagonal channels and selective Cu substitution only on two out of seven Pb sites. At *x* > 0.5 in Pb_10–*x*_Cu_*x*_(PO_4_)_6_O, Cu cations are introduced onto all Pb sites, which triggers the transition to the archetypical apatite structure, reflecting the coupling of the core structural components of the apatite framework in the ordering pattern.

## Introduction

The report of room temperature superconductivity in a material labeled LK-99 has attracted worldwide public interest.^1^ The material is a complex mixture containing Pb_10-x_Cu_*x*_(PO_4_)_6_O, Cu and Cu_2_S and potentially other compounds, which was reported to show levitation above a permanent magnet and a decrease in resistance by several orders of magnitude at 127 °C.^2^ Subsequent reports have not confirmed room-temperature superconductivity and the drop in resistance has been attributed to the superionic phase transition of Cu_2_S.^3,4^

Pb_10-x_Cu_*x*_(PO_4_)_6_O – the main component in LK-99 – belongs to the family of apatites.^4−6^ Apatites are considered for applications in catalysis, immobilization of radioactive waste and heavy metals and ceramic membranes based on chemical flexibility and microporous channel structure.^7^ However, apatites play the most important role wherever they occur naturally either in minerals as source for phosphate used in fertilizers or as the inorganic component in bone mineral.^7^ Although oxide ion conductivity has been observed, apatites are generally electronic insulators. On the other hand, several complex lead containing cuprates such as in Pb_2_Sr_2_(Y,Ca)Cu_3_O_8+δ_ or (Pb,Cu)(Sr,La)_2_CuO_5−δ_ are high-temperature superconductors.^8,9^ Revealing the location of the copper cations in the apatite framework and their influence on that framework will clarify the structure–property relationships in this family of materials containing LK-99.

**Figure 1:**
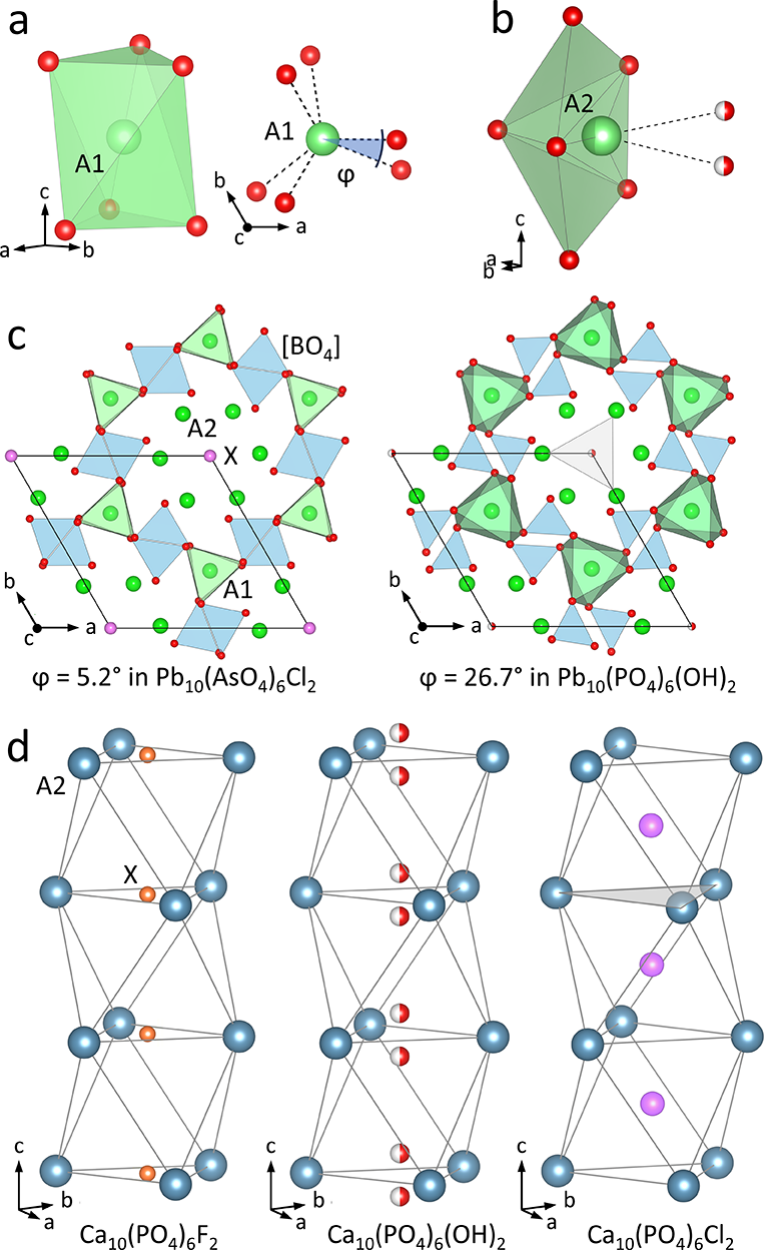
Structural features of representative apatites in space group *P*6_3_/*m*. (a) Perspective view and top-down view of the trigonal metaprismatic coordination of the A1 site with twist angle ϕ. (b) Perspective view of the distorted pentagonal pyramidal coordination of the A2 site with split site for the X anion as observed in Ca_10_(PO_4_)_6_OH_2_ apatites. (c) Projection of Pb_10_(AsO_4_)_6_Cl_2_ and Pb_10_(PO_4_)_6_(OH)_2_ apatites along (001) emphasizing the flexible framework of A1 metaprisms and [BO_4_] tetrahedra around the 1D channels filled by X anions. The two examples exhibit a large difference in ϕ. (d) Positions of different X anions shown in panel (b) demonstrating their position in the A2 octahedra of the channels of Ca_10_(PO_4_)_6_X_2_ apatites. The channels are formed by face-sharing Ca-octahedra with the channel cross section shown as a gray triangle. Hydrogen omitted for clarity. Ca^2+^ cations in blue, Pb^2+^ in green, F^–^ in orange, O^2–^ in red, and Cl^–^ in pink.

The apatite family is a large class of minerals of the general composition (A1)_4_(A2)_6_(BO_4_)_6_X_2-y_.^10^ Apatite structures are characterized by [BO_4_] tetrahedra, which are connected via the trigonal (meta-)prisms of the A1-cation forming a 3D framework with 1D channels parallel to [001] (see Figure 1a,c). This robust framework can accommodate a large variety of A1 and A2 cations including alkali, alkaline earth, or rare-earth cations, such as Na^+^, Ca^2+^, La^3+^ and Pb^2+^. Most commonly, these minerals are phosphates or silicates, but other penta- and tetravalent B cations have also been reported, including Ge^4+^, V^5+^ and As^5+^.^10^ The [BO_4_] tetrahedra coordinate the A2 site in a distorted pentagonal pyramidal coordination with the A2 site approximately in the pentagonal face (see Figure 1b). The X anion site(s) complete the coordination environment of the A2 site. The pentagonal faces form infinite 1D channels lined with the A2 cations. The A2 cations themselves are arranged in stacks of face-sharing octahedra containing the X anions along the center axis. The channels can act as diffusion pathways for these anions. X can be F^–^, Cl^–^, Br^–^, (OH)^−^ and O^2–^ anions with *y* ≤ 2 depending on the valence of the ions. Vacancies in those channels can result in anion mobility.^11^

The location of the X anions inside the 1D channels varies depending on the radius of the anion (see Figure 1d) and the cross section of the channel. The minimal channel cross section occurs at the equilateral A2 triangle, which is the shared triangular face between the A2 octahedra stacked along the *c* axis.^10^ The large Cl^–^ anions in Ca_5_(PO_4_)_3_Cl-type apatite structures are located in the center of the Ca octahedra lining the 1D channels.^12^ In contrast, the small F^–^ anions in the Ca_5_(PO_4_)_3_F-type structures reside in the triangular faces of the Ca-octahedra.^13^ For intermediate size anions, Ca_5_(PO_4_)_3_(OH)-type apatites are observed, in which the anions are displaced from the triangular faces due to short Ca-X distances.^14^

In addition to the simple F^–^, Cl^–^, Br^–^, and O^2–^ anions, polyatomic anions can be incorporated in the hexagonal channels of the apatite structure. In Ca_9.75_[(PO_4_)_5.5_(CO_3_)_0.5_]CO_3_ one of the three C–O bonds of the carbonate group points along the channel axis,^15^ whereas Sr_10_[(PO_4_)_5.5_(BO_4_)_0.5_](BO_2_) contains linear [BO_2_]^−^ anions pointing along the along the channel axis.^16^ Moreover, several studies report apatites with linear [CuO]^−^ chains filling the hexagonal channels.^17−19^ The presence of these chains causes the unit cell to expand in the *ab*-plane compared to the analogous parent compound without [CuO]^−^ chains. The possibility of Cu in the hexagonal channels needs to be considered when the structure of the apatite component in LK-99 is investigated.

The majority of apatites (ca. 60%) have been described in the hexagonal space group *P*6_3_/*m* (no. 176), but several lower symmetry variants (among others *P*6_3_, 21%, and *P*3̅, 9%) have been reported.^10^ The monoclinic space group *P*2_1_/*b* with a doubled *b* lattice parameter is common for [OH]^−^ rich apatites as a result of the [OH]^−^ groups ordering over the aforementioned split position to avoid short H–H contacts.^20,21^ (The reference databases used in this study are provided in the Supporting Information.) The archetypical apatites in *P*6_3_/*m* that follow the general chemical formula A1_4_A2_6_(BO_4_)_6_X_2-y_ have a unit cell volume of 422 Å^3^ < V_uc_ < 758 Å^3^ highlighting the flexibility of the structural framework.^22^ The lower bound was reported for Mg_5_(PO_4_)_3_(OH), whereas the upper limit is encountered in Ba_5_(VO_4_)_3_Br.^23,24^ The *c*/*a* ratio falls in the range of 0.71 < *c*/*a* < 0.75 for the vast majority of these compounds.^22^ Exceptions occur whenever the ratio between the size of [BO_4_] tetrahedra and the A cation radius drastically differs such as in Ba_5_(VO_4-x_S_*x*_)_3_Cl which is *P*6_3_/*mcm* with *c*/*a* = 0.84,^25^ or Mn_5_(AsO_5_)_3_Cl which is *P*6_3_/*m* with *c*/*a* = 0.63.^26^

Among the phosphate apatites, NaBa_3_La(PO_4_)_3_F-type and the Ca_10_(PO_4_)_6_O-type structures are the only ones that crystallize in space group *P*6̅ (no. 174).^27,28^ Importantly, both structures have the same unit cell size as the apatite archetype. The lower space group symmetry in these structures is a consequence of ordered site decoration patterns of the A1 or X sites. In addition to several low symmetry variants with equivalent unit cell size, several modulated structures have been documented. Most of these modulated structures exhibit ordering in the *ab* plane. This includes the aforementioned hydroxyapatites with doubled *b* lattice parameter as well as Ba_5_(VO_3_S)_3_F-type and Ba_10_(VO_3_S)_6_S-type structures, which form 2 × 2 × 1 superstructures in *P*3̅*m*1 (no. 164) and *P*6_3_ (no. 173), respectively.^25,31^ In both Ba_5_(VO_3_S)_3_F-type and Ba_10_(VO_3_S)_6_S-type structures, the *a*- and *b*-lattice parameters are doubled because of alternating X-vacancy ordering in the *ab*-plane. Furthermore, incommensurate modulation in the *ab*-plane has been reported in halogen deficient Cd_5_(PO_4_)_3_Br and Cd_5_(VO_4_)_3_I.^32^ Conversely, Ca_15_(PO_4_)_9_IO-type and La_3_Nd_11_(SiO_4_)_9_O_3_-type structures exhibit commensurate ordering along the *c* direction resulting in 1 × 1 × 3 superstructures in *P*6_3_/*m*. The former is characterized by two aliovalent anions in the 1D channels alternating with vacancies along the *c* direction, the latter shows ordering of the La^3+^ and Nd^3+^ cations on the A1 site along the *c* direction.^29,30^ Lastly, incommensurate ordering along the *c* direction has been reported in the metal deficient La_9.33_(GeO_4_)_6_O_2_ apatites along the *c*-direction.^33^

We present the crystal structure of Pb_10-x_Cu_*x*_(PO_4_)_6_O (*x* < 0.5) with a unique 1 × 1 × 2 superstructure of the apatite archetype distinct from all previously reported apatite superstructures. Our investigations reveal that Pb_10-x_Cu_*x*_(PO_4_)_6_O with *x* < 0.5 crystallizes in space group *P*6̅ with coupled anion and cation ordering. Lastly, we explain the mechanisms driving the transformation to the higher symmetry structure of the archetype (see Figure 1) for Pb_10-x_Cu_*x*_(PO_4_)_6_O with *x* > 0.5.

## Results and Discussion

### Synthesis and Powder Diffraction

During the synthesis which targeted the nominal composition Pb_9.5_Cu_0.5_(PO_4_)_6_O (see the Experimental Section below), the reactants melted, and the crystalline, gray products formed a thin layer at the bottom of the alumina crucible. The powder diffraction pattern of the sample can be indexed based on monoclinic Pb_3_(PO_4_)_2_ (ICSD coll. code 66379) and apatite Pb_10_(PO_4_)_6_O (*P*6_3_/*m*, ICSD coll. code 98703). Pb_3_(PO_4_)_2_ forms due to the compositional proximity of the starting mixture to the peritectic reaction 1) in the binary PbO-P_2_O_5_ system:^5^
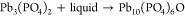
1

Refinement of the synchrotron powder X-ray diffraction data (see Figure S1) provided the lattice parameters of *a* = *b* = 9.8335(2) Å and *c* = 7.4224(2) Å for the apatite phase. These lattice parameters are slightly smaller than the published lattice parameters of *a* = *b* = 9.8650(3) Å and *c* = 7.4306(3) Å for Pb_10_(PO_4_)_6_O,^34^ which is consistent with partial substitution of Pb^2+^ by the smaller Cu^2+^ cations (r_Pb_ = 1.19 Å, r_Cu_ = 0.73 Å).^35^ Refining the mixed occupancies of the A1 and A2 sites yields the composition Pb_8.66(5)_Cu_1.33(5)_(PO_4_)_6_O_2_. The sample consists of 79(1) wt % oxyapatite and 20.7(4) wt % Pb_3_(PO_4_)_2_.

### Single Crystal Diffraction and Crystal Structures

Several single crystals (see the Experimental Section below for details on the synthesis) were screened during in-house single crystal diffraction experiments and seven full data sets were collected before the best crystals were sent to beamline I19 at Diamond Light Source. The screening revealed two groups of hexagonal crystals with distinct cell volumes in addition to a few crystals of monoclinic Pb_3_(PO_4_)_2_. One group of hexagonal crystals exhibits a diffraction pattern consistent with the apatite archetype in space group *P*6_3_/*m* and a unit cell volume of roughly V_uc_ ≈ 610 Å^3^, whereas the unit cell for second set of hexagonal crystals in space group *P*6̅ has a volume of 1232 Å^3^ and thus approximately twice the unit cell volume of the apatite archetype. Such a doubling of the unit cell has been listed in a single table entry by *Engel et. al*. for Pb_10_(PO_4_)_6_O (V = 2 × 621.3 Å^3^) without further comments on the structure, and no structure solution is presented.^6^ In the following, we will first briefly discuss the refined single crystal structure of the crystals with smaller unit cells as they belong to a solid solution Pb_10-x_Cu_*x*_(PO_4_)_6_O with *x* > 0.5, before presenting the complex superstructure of the crystals with a larger unit cell.

The synchrotron single crystal diffraction data of Pb_10-x_Cu_*x*_(PO_4_)_6_O with *x* > 0.5 can be indexed to a hexagonal unit cell with lattice parameters *a* = *b* = 9.78150(3) Å, and *c* = 7.35600(3) Å (V = 609.513(4) Å^3^) in space group *P*6_3_/*m* (no. 176). All cation sites and several oxygen sites are identified when the structure is initially solved. The remaining anion sites appear as peaks in the electron density difference map after a few refinement cycles. All atoms are refined with anisotropic displacement parameters except for two partially occupied O sites. The sum of the occupancies of the latter has been constrained to one O^2–^ per formula unit to maintain charge neutrality.

**Figure 2:**
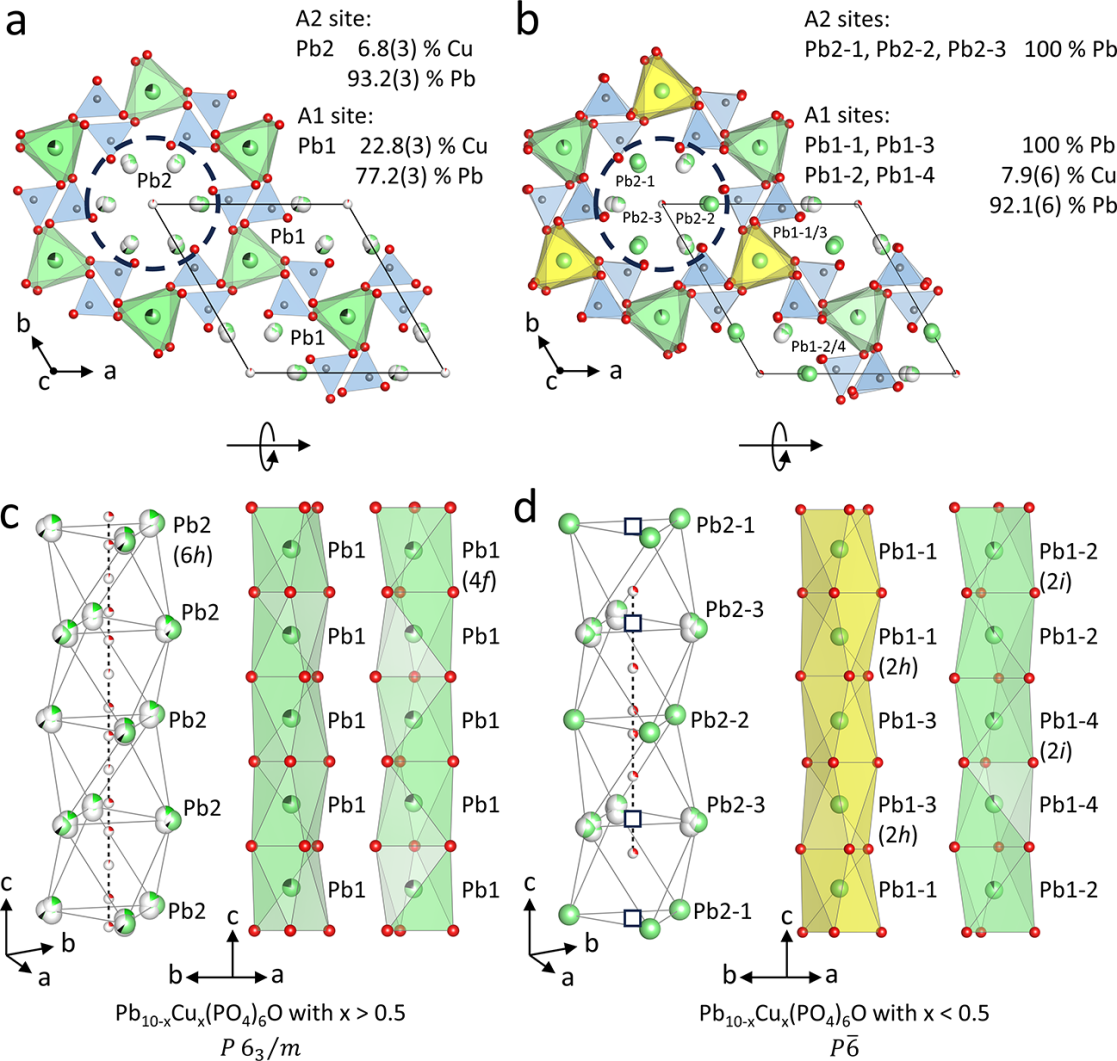
Projections of the crystal structures of the solid solutions Pb_8.68(3)_Cu_1.32(2)_(PO_4_)_6_O (*x* > 0.5) in *P*6_3_/*m* (a) and Pb_9.842(8)_Cu_0.158(8)_(PO_4_)_6_O (*x* < 0.5) in P6̅ (b). Perspective views of the respective 1D channels and side views of the stacks of trigonal metaprisms as the two key motifs are shown in panels (c) and (d). The dashed circles in panels (a) and (b) highlight the 1D channels. The stacks of trigonal metaprisms shown in green contain mixed occupied sites; yellow trigonal metaprisms contain sites exclusively occupied by Pb. The stack of metaprisms labeled Pb1–2/4 in panel (b) contains the sites Pb1–2 and Pb1–4, while the stack of metaprisms labeled Pb1–1/3 contains the sites Pb1–1 and Pb1–3 as seen in panel (d). Boxes indicate the location of vacancies in the O^2–^ ordering. [PO_4_]^3–^ tetrahedra are shown in blue, Pb^2+^ cations are light green, Cu^2+^ is black, P^5+^ is gray, and O^2–^ is red.

The structure of Pb_10-x_Cu_*x*_(PO_4_)_6_O where *x* > 0.5 is closely related to the apatite archetype structure (Figure 1). The metal cations reside in trigonal metaprisms (Pb1, Wyckoff site 4*f*) and distorted pentagonal pyramids (Pb2, Wyckoff site 6*h*) (see Figure 2a) formed by the oxygen atoms of the phosphate group resulting in a 3D framework with 1D channels running along [001]. It has been shown that the unit cell volume increases with increasing amount of Cu in the hexagonal channels,^17^ while it decreases with Cu substituting the cations in the A-sites.^36^ The unit cell volume of Pb_10-x_Cu_*x*_(PO_4_)_6_O is smaller than the unit cell volume of the parent Pb_10_(PO_4_)_6_O, inferring that Cu^2+^ is not located in the 1D channels, but instead partially substitutes at the Pb^2+^ position. The metal sites in the solid solution are labeled as Pb1 and Pb2 and both sites show mixed occupancies. The Pb1 site is occupied by 77.2(3)% Pb and 22.8(3)% Cu, while the Pb2 site is occupied by 93.3(2)% Pb and only 6.8(3)% Cu. Thus, the composition for this single crystal is Pb_8.68(3)_Cu_1.32(2)_(PO_4_)_6_O (*x* > 0.5). The distinct preferential occupancy of the Pb2 site by Pb has been observed in several lead apatite solid solutions.^4,6,37,38^ In general, larger cations favor the A2 site because of its higher coordination number (CN = 6 for A1 and CN = 7 for A2, respectively).^10^ Additionally, it has been shown that Pb^2+^ preferentially occupies the A2 site when paired with cations of similar radius such as Ca.^35^ This has been attributed to the lower site symmetry and the partially empty channels being favorable for Pb^2+^ with its stereoactive electron lone pair.^38^

**Figure 3:**
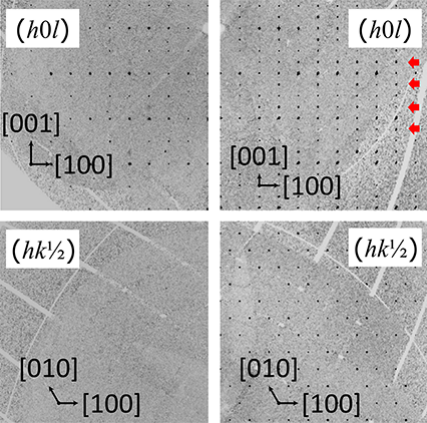
Precession images of Pb_8.68(3)_Cu_1.32(2)_(PO_4_)_6_O (*x* > 0.5, left) and Pb_9.842(8)_Cu_0.158(8)_(PO_4_)_6_O (*x* < 0.5, right). The superstructure reflections in the (h0l) plane are indicated by red arrows. The () plane (based on the subcell) shows the absence of superstructure reflections for Pb_8.68(3)_Cu_1.32(2)_(PO_4_)_6_O.

The Pb1 site is coordinated in a trigonal metaprismatic fashion by six oxide anions from adjacent phosphate groups. The metaprisms are stacked along the *c*-direction sharing trigonal faces. The twist angle ϕ for the trigonal metaprisms is ϕ = 20.4(3)° for Pb_8.68(3)_Cu_1.32(2)_(PO_4_)_6_O (*x* < 0.5), which is in the commonly observed range of 5° < ϕ < 25° for apatites.^39^ The rigid phosphate groups coordinate both the Pb1 and Pb2 sites effectively coupling the twist angle to the channel cross section.

**Figure 4:**
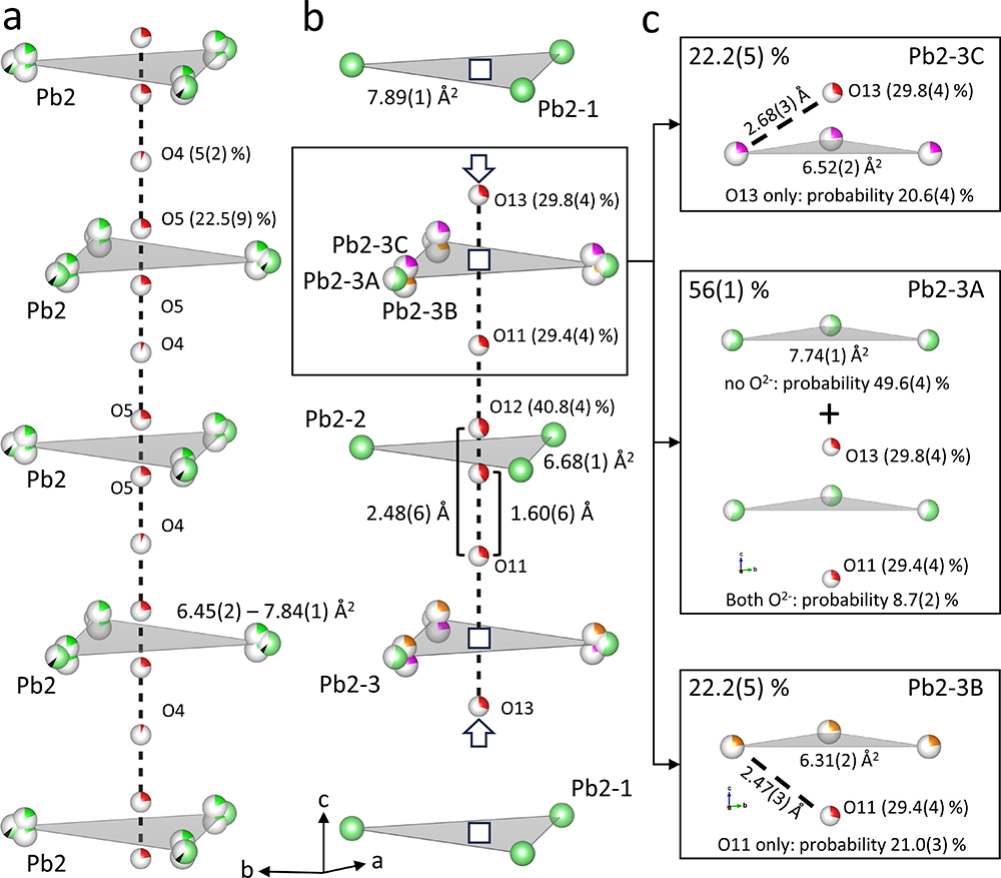
Perspective views of the channels in Pb_10-x_Cu_*x*_(PO_4_)_6_O with *x* > 0.5 (a) and Pb_10-x_Cu_*x*_(PO_4_)_6_O with *x* < 0.5 (b) highlight the cross-sectional area (light gray) of the 1D channels. Boxes represent vacancies, and arrows show displacement of the O^2–^ anions in panel ( b). The superposition of the split Pb2–3 site is resolved in panel (c) with four configurations with the corresponding probabilities for the nearby O^2–^ anions and the triangular channel cross-section. Cu^2+^ in black, O^2–^ in red, Pb^2+^ in green with the split Pb2–3B sites in orange, and Pb2–3C in pink, respectively.

Further refinement of the initial structure solution reveals splitting of the pentagonal pyramidal Pb2 site, and the presence of two distinct, partially occupied O^2–^-sites in the 1D channels. The O^2–^ anion sites are located at (0, 0, 0.176(3)) and (0, 0, ) and correspond to O5 (22.5(9)% occupancy) and O4 (5(2)% occupancy), respectively. The more frequently occupied O5 site is split due to its displacement from the center of the Pb2 triangle as the shared face of the Pb2 octahedra. The O4 site is located in the center of an octahedron formed by Pb2 (see Figure 4a). This anion configuration in the channels of Pb_10-x_Cu_*x*_(PO_4_)_6_O with *x* > 0.5 represents a combination of the X anion arrangements in Ca_10_(PO_4_)(OH)_2_ (see Figure 1c, middle) and Ca_10_(PO_4_)(Cl)_2_ (see Figure 1c, right) with O^2–^ predominantly residing on a split site around the center of the shared octahedral face.

The disorder in the 1D channels also induces positional disorder at the nearby Pb2 site. The Pb2 site is split into one main site (Pb2) and two equivalent satellite sites (Pb2S). The occupancies of the split A2 site (64.2(4)% for Pb2 and 17.9(1)% for Pb2S) correlate well with the probability of neither of the adjacent O5 sites being occupied (60(1)% probability) or just one of them being occupied (20.8(7)% probability). This demonstrates that the presence or absence of an oxide anion at the O5 site affects the position of the nearby metal cation. The disordered anions and the split Pb2 site are akin to the positional disorder encountered in Pb_9.85_(VO_4_)_6_I_1.7_, where disordered I^–^ causes positional disorder on the Pb-filled A2 site.^40^

The second group of crystals is indexed with a hexagonal unit cell in the noncentrosymmetric space group *P*6̅ (no. 174), which is an uncommon space group symmetry among apatites. Compared to Pb_8.68(3)_Cu_1.32(2)_(PO_4_)_6_O and other single crystals with Pb_10-x_Cu_*x*_(PO_4_)_6_O with *x* > 0.5, the diffraction pattern indicates almost identical *a* lattice parameters, but displays additional superstructure reflections at (0, 0, ) at both 100 and 300 K (see Figure 3) which doubles the unit cell volume. The single crystal synchrotron diffraction data for a crystal with the composition Pb_9.842(8)_Cu_0.158(8)_(PO_4_)_6_O (*x* < 0.5) confirm the hexagonal unit cell in the noncentrosymmetric space group *P*6̅ (no. 174) with *a* = *b* = 9.80260(4) Å and *c* = 14.80060(2) Å. As for Pb_8.68(3)_Cu_1.32(2)_(PO_4_)_6_O, the smaller *a* lattice parameter compared to Pb_10_(PO_4_)_6_O is evidence that Cu^2+^ substitutes Pb^2+^ instead of being incorporated in the hexagonal channels.^6^ Solving the data as an inversion twin revealed all cation sites and approximately half of the anion sites. The remaining O^2–^ sites emerged as peaks in the electron density difference map after a few refinement cycles. The metal cations were refined using anisotropic displacement parameters, whereas the light elements P and O are refined with isotropic displacement parameters due to the large difference in electron density between Pb and the other elements. The results of the refinement are summarized in Table 1.

**Table 1:** Details of the Single-Crystal Refinements

Chemical formula	Pb_9.842(8)_Cu_0.158(8)_(PO_4_)_6_O	Pb_8.68(3)_Cu_1.32(2)_(PO_4_)_6_O
Formula weight [g/mol]	2634.74	1234.05
Crystal system, space group	hexagonal, *P*6̅, no. 174	hexagonal, *P*6_3_*/m*, no. 176
Lattice parameters	*a* = *b* = 9.80260(4) Å	*a* = *b* = 9.78150(3) Å
	*c* = 14.80060(2) Å	*c* = 7.35600(3) Å
	α = β = 90°, γ = 120°	α = β = 90°, γ = 120°
Volume [Å^3^], Z	1231.664(14), 2	609.513(4), 2
Density [g/cm^3^]	7.104	6.724
Temperature [K]	100(2)	100(2)
Absorption coefficient [mm^–1^]	62.447	56.638
*F* (000)	2203	1040
Crystal size [mm^3^]	0.070 × 0.043 × 0.034	0.042 × 0.040 × 0.033
Θ range [°]	1.334 to 35.991	2.330 to 36.037
*hkl* ranges	–16 ≤ *h* ≤ 16,	–16 ≤ *h* ≤ 16,
	–16 ≤ *k* ≤ 15,	–16 ≤ *k* ≤ 16,
	–24 ≤ *l* ≤ 24	–12 ≤ *l* ≤ 12
Reflections (collected/independent/R_int_)	27259/4227/0.0663	12536/1021/0.0381
data/restraints/parameters	4227/1/97	1021/1/48
Goodness-of-fit on *F*^2^	1.070	1.243
Final *R* indices (I > 2σ(I))	*R*_1_ = 0.0266, *wR*_2_ = 0.0708	*R*_1_ = 0.0182, *wR*_2_ = 0.0382
*R* indices (all data)	*R*_1_ = 0.0324, *wR*_2_ = 0.0725	*R*_1_ = 0.0207, *wR*_2_ = 0.0387
Extinction coefficient	0.00024(4)	0.00105(10)
Largest diff. Peak/hole [e^–^/Å^3^]	5.484/–3.353	2.854/–1.524

The structure of Pb_10-x_Cu_*x*_(PO_4_)_6_O with *x* < 0.5 contains seven metal sites. The larger number of crystallographically independent sites is a consequence of the larger unit cell and lower symmetry compared to Pb_10-x_Cu_*x*_(PO_4_)_6_O with *x* > 0.5. There is a set of four A1 sites (Pb1–1, Pb1–2, Pb1–3 and Pb1–4) located in trigonal metaprisms, which are stacked in two symmetry-independent columns along the crystallographic *c*-direction. Of these four sites, two are Wyckoff sites 2*h* (Pb1–1 and Pb1–3), while the other two sites (Pb1–2 and Pb1–4) are Wyckoff sites 2*i*. Remarkably, only the 2*i* sites (Pb1–2 and Pb1–4) in one of the two stacks are both partially (7.9(6)%) occupied by Cu. The four trigonal metaprisms vary in height from 3.63(1) to 3.78(1) Å and twist angle ϕ (see Figure 1a and Figure S4). Notably, the largest twist angle ϕ = 24.5(7)° occurs for the metaprisms centered at Pb1–2 and the smallest twist angle (ϕ = 18.4(8)°) for Pb1–4 at the other 2*i* site, whereas the twist angles for Pb1–1 and Pb1–3 sites are 20.8(6)° and 22.7(6)°, respectively.

The set of three A2 sites (Pb2–1 (3*j*), Pb2–2 (3*k*) and Pb2–3 (6*l*)) make up the hexagonal channels along [001]. The lead cations of the A2 sites are in a disordered coordination environment. The oxygen atoms of the phosphate groups coordinate the A2 sites in a distorted pentagonal pyramidal geometry, while the partially disordered O anions in the 1D channels complete the coordination environments. Each of the three sites Pb2–1, Pb2–2 and Pb2–3, forms an equilateral triangle that defines the cross section of the 1D channels. These triangles occur in the sequence Pb2–3, Pb2–2, Pb2–3 and Pb2–1 (see Figure 4b). Importantly, the lateral positions of Pb2–1, Pb2–2 and Pb2–3 are not identical. All three of the A2 sites in Pb_10-x_Cu_*x*_(PO_4_)_6_O with *x* < 0.5 are exclusively occupied by lead, reflecting the higher coordination number of the A2 sites compared to the A1 sites. The electron density map (Figure S3) shows that the electron density around the Pb2–3 site is not ellipsoidal, which indicates a split site. The split site is modeled with three partially occupied sites Pb2–3A, Pb2–3B and Pb2–3C.‡ The sum of the occupancies was constrained to 100% during the structural refinement. The split Pb2–3 site and the corresponding occupancies resemble the split Pb2 site in Pb_10-x_Cu_*x*_(PO_4_)_6_O with *x* < 0.5 described above.

The rigid phosphate groups link the A1 trigonal metaprisms with the A2 Pb cations forming the channels. The O^2–^ anions in the channel are identified by inspecting the electron density map which shows three O^2–^ sites as small peaks at distinct (0,0,*z*) positions in the 1D channels. The occupancies of the three sites O11, O12 and O13 (all Wyckoff sites 2*g*) are constrained to maintain charge balance.

The site O12 is split around the center of the Pb6 triangle and is 40.8% occupied. This motif closely resembles the arrangement of Pb2 and O5 sites in Pb_10-x_Cu_*x*_(PO_4_)_6_O with *x* > 0.5. However, in Pb_10-x_Cu_*x*_(PO_4_)_6_O with *x* < 0.5 only the Pb2–2 triangle is associated with a split O12 site out of the set of three A2 sites, whereas every Pb2 triangle in Pb_10-x_Cu_*x*_(PO_4_)_6_O with *x* > 0.5 is associated with a split O5 site.

The sites O11 and O13 (occupancies 29.4(4)% and 29.8(4)%, respectively) are both located in A2 octahedra. However, both O^2–^ anions are shifted off the center of their respective A2 octahedra toward the Pb2–3 site.

The O^2–^ arrangement in the channels of Pb_10-x_Cu_*x*_(PO_4_)_6_O with *x* < 0.5 is a combination of the X anion configurations observed in Ca_10_(PO_4_)(OH)_2_ (see Figure 1c, middle) and Ca_10_(PO_4_)(Cl)_2_ (see Figure 1c, right) with anion vacancy ordering. The octahedral sites are partially filled by O11 and O13 similar to Ca_10_(PO_4_)(Cl)_2_. A split O^2–^ site analogous to Ca_10_(PO_4_)(OH)_2_ occurs at every fourth Pb triangle (Pb2–2, see Figure 4b). This arrangement of oxide anions in the apatite channels is unique and is responsible for doubling of the *c*-lattice parameter.

**Figure 5:**
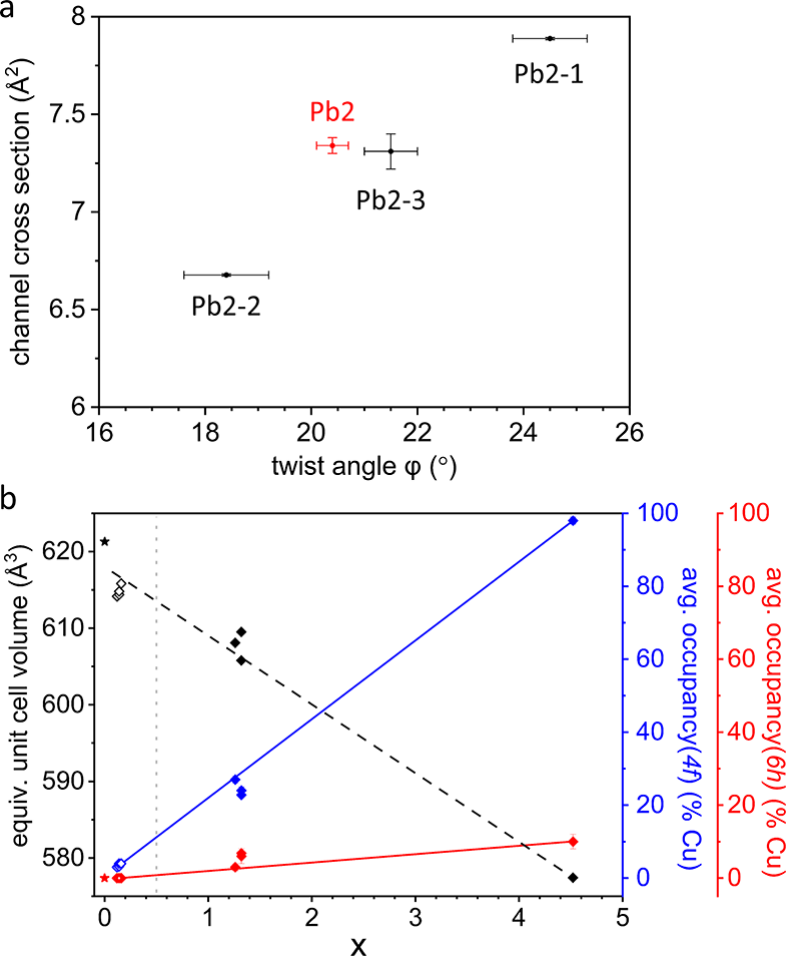
(a) Weighted average of the channel cross section area plotted against the average twist angle of the coupled mixed occupied metaprisms for Pb_8.68(3)_Cu_1.32(2)_(PO_4_)_6_O (*x* > 0.5, red) and Pb_9.842(8)_Cu_0.158(8)_(PO_4_)_6_O (*x* < 0.5, black) showing correlation between twist angle and channel cross section. (b) Unit cell volume (black) and occupancies of the two metal sites in the apatite solid-solution Pb_10-*x*_Cu_*x*_(PO_4_)_6_O. The average occupancy of the metal site(s) with trigonal prismatic coordination in blue and the average occupancy of metal site(s) forming the 1D channels in red. The filled diamond symbols indicate the archetypical unit cell in *P*6_3_/*m*; open symbols signify a 1 × 1 × 2 supercell in P6̅. Stars denote Pb_10_(PO_4_)_6_O reported at room temperature by Engel et al.^6^ The dotted gray line marks the starting composition. The straight lines are a guide to the eye.

In essence, there are five structural features beyond the unit cell size and space group symmetry that distinguish Pb_10-x_Cu_*x*_(PO_4_)_6_O with *x* < 0.5 from the archetypal apatite Pb_10-x_Cu_*x*_(PO_4_)_6_O with *x* > 0.5. These are (I) the site decoration pattern of the lead sites, (II) the distinct twist angles for each of the four A1 sites, (III) the unequal lateral positions of the A2 cations, (IV) the oxygen ordering in the 1D channels and (V) Pb2–3 being the only split site among the A2 sites. These will be discussed in detail in the following sections.

(I) The lower symmetry of Pb_10-x_Cu_*x*_(PO_4_)_6_O with *x* < 0.5 arises from the selective substitution of Pb^2+^ by Cu^2+^ on only two out of seven Pb-sites (Pb1–2 and Pb1–4), both of which belong to the set of A1 sites located in the same stack of twisted trigonal metaprisms: Cu is not found on the three A2 sites forming the 1D channel in the superstructure. (II) The trigonal metaprisms in the stack of the Pb1–2 and Pb1–4 sites (both Wyckoff site 2h) also exhibit the largest differences in the twist angle compared to Pb_10-x_Cu_*x*_(PO_4_)_6_O with *x* > 0.5. The twist angle for the Pb1–2 metaprism is ϕ = 24.5(7)° occurs and the twist angle (ϕ = 18.4(8)°) for the Pb1–4 metaprism at the other 2*h* site, whereas the twist angles for Pb1–1 and Pb1–3 sites are 20.8(6)° and 22.7(6)°, respectively. In comparison, the metaprism twist angle in Pb_10-x_Cu_*x*_(PO_4_)_6_O with *x* > 0.5 is ϕ = 20.4(3)°. The twist angle ϕ commonly ranges from 15° to 25°^39^ and it is very sensitive to the characteristics of the species occupying these sites, such as ionic radii, site occupancy etc.^39^ Therefore, it is reasonable to assume that the site decoration pattern is linked to these differences in twist angles.

(III) The three A2 sites (Pb2–1, Pb2–2 and Pb2–3) each form an equilateral triangle. These triangles correspond to the minimal cross section of the 1D channels. As the lateral positions (in the *ab*-plane) of the three sites differ slightly from each other (see Table S1), the area of the three equilateral triangles defined by the Pb2–1, Pb2–2 and Pb2–3 sites are different as well. This results in a modulated cross section of the 1D channels in the 1 × 1 × 2 superstructure, in contrast to the constant cross section in the archetype (see Figure 4). It has been shown that the channel cross section in apatites is inversely related to the twist angle ϕ since the A1 and A2 sites are coupled via the rigid phosphate groups.^39^ This is also the case for Pb_10-x_Cu_*x*_(PO_4_)_6_O with *x* < 0.5: Figure 5a shows, that the weighted average of the channel cross section correlates well with the average twist angle of the connected metaprisms. Thus, the distinct twist angles of the four metaprisms in Pb_10-x_Cu_*x*_(PO_4_)_6_O with *x* < 0.5 are coupled to the modulated channel cross sections along the *c*-direction (see Figure 4b) and vice versa.

The largest channel cross section occurs for the Pb2–1 triangle and the smallest channel cross section is observed for Pb2–2. In the sequence Pb2–3, Pb2–2, Pb2–3 and Pb2–1, the cross section of the 1D channel expands and contracts along its length and couples with the position of the O^2–^ in the channels, which are more ordered in the modulated channel of Pb_10-x_Cu_*x*_(PO_4_)_6_O with *x* < 0.5 than the disordered O^2–^ in the uniform channels of Pb_10-x_Cu_*x*_(PO_4_)_6_O with *x* > 0.5.

(IV) While the O^2–^ anion arrangement in Pb_10-x_Cu_*x*_(PO_4_)_6_O with *x* > 0.5 represents a completely disordered combination of the X anion motifs in Ca_10_(PO_4_)_6_(OH)_2_ and Ca_10_(PO_4_)_6_(Cl)_2_, the O^2–^ arrangement in Pb_10-x_Cu_*x*_(PO_4_)_6_O with *x* < 0.5 resolves some of this disorder to create a superstructure where the two motifs are combined with anion vacancies at two of the three sets of A2 triangles (Pb2–1 and Pb2–3). Pb_10-x_Cu_*x*_(PO_4_)_6_O with *x* > 0.5 exhibits a split O5 site for every A2 triangle in the channel, whereas the split O12 site in Pb_10-x_Cu_*x*_(PO_4_)_6_O with *x* < 0.5 occurs only for the Pb2–2 triangle, which forms every fourth Pb triangle along the channels. Another difference from the structures of Pb_10-x_Cu_*x*_(PO_4_)_6_O with *x* > 0.5 and Ca_10_(PO_4_)_6_Cl_2_ (see Figures 4a and 1c, respectively) is that the anion sites O11 and O13 in Pb_10-x_Cu_*x*_(PO_4_)_6_O with *x* < 0.5 are not perfectly located in the center of the corresponding Pb octahedron. This in turn results in a long distance between O13 and Pb3 (d = 3.47(1) Å) and thus a large channel cross section due to the electrostatic repulsion between the Pb2–1 cations (see Figure 4b).

(V) The final distinguishing feature of Pb_10-x_Cu_*x*_(PO_4_)_6_O with *x* < 0.5 is the split Pb4 site. While every A2 site in Pb_10-x_Cu_*x*_(PO_4_)_6_O with *x* > 0.5 is split, Pb2–3 is the only split site out of the set of three A2 sites in Pb_10-x_Cu_*x*_(PO_4_)_6_O with *x* < 0.5. We note that one could expect splitting of the Pb2–2 site based on its coordination environment being analogous to the coordination environment of the split Pb2 site in Pb_10-x_Cu_*x*_(PO_4_)_6_O with *x* > 0.5. Indeed, the anisotropic thermal displacement parameters for Pb2–2 are notably larger than for the other lead sites in Pb_10-x_Cu_*x*_(PO_4_)_6_O with *x* < 0.5, which could indicate such a displacement, but the data do not allow the refinement as a split site.

Coming back to the split Pb2–3 site, its coordination environment is unique in that the partially occupied O11 and O13 sites are significantly closer than the two partially occupied O13 around Pb2–1 and in the absence of the split O12 site. The close proximity of the partially occupied O11 and O13 sites leads to four possible O^2–^ configurations adjacent to Pb2–3:1) neither O11 nor O13 occupied, 2) only O11 occupied, 3) only O13 occupied, and 4) both O11 and O13 occupied (see Figure 4c). The different oxygen environments affect the position of the Pb2–3 cation causing its site to split. The probabilities for these oxygen configurations agree well with the occupancies of the Pb2–3B and Pb2–3C sites (see Figure 4c). This demonstrates clearly that the channel diameter correlates with the oxygen positions. Similarly split sites for Ba and Cl have been observed in the solid solution series Ba_5_(VO_4-δ_S_δ_)_3_Cl with δ = 0.966, where disorder in the Ba^2+^ position is introduced by the nearby Cl^–^ anion disorder.^25^

**Figure 6:**
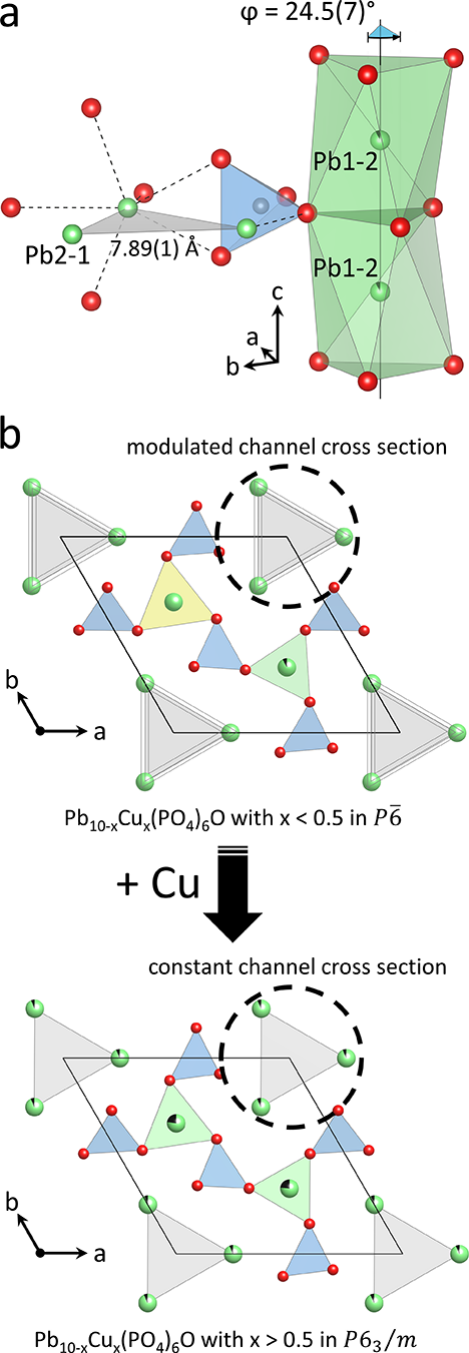
(a) Perspective view of the [PO_4_]^3–^ group coupling the twisted trigonal metaprisms to the 1D channels in in Pb_10-*x*_Cu_*x*_(PO_4_)_6_O with *x* < 0.5. (b) Schematic illustration of how the incorporation of Cu causes the structural transformation into Pb_10-*x*_Cu_*x*_(PO_4_)_6_O with *x* > 0.5, which is accompanied by a transition from a modulated to a constant channel cross section (dashed circles) and thus changing the oxygen ordering in these channels. [PO_4_]^3–^ tetrahedra are shown in blue, Pb^2+^ cations are light green, Cu^2+^ is black, O^2–^ is red, and the channel cross section is in light gray.

To summarize, Pb_10-x_Cu_*x*_(PO_4_)_6_O with *x* < 0.5 crystallizes in a lower symmetry 1 × 1 × 2 superstructure of the apatite archetype structure observed for Pb_10-x_Cu_*x*_(PO_4_)_6_O with *x* > 0.5. The observed O^2–^ ordering and the site decoration pattern of the Pb sites are in concert with the modulated channel cross section and the twist angles of the metaprisms, respectively. The twist angles and the channel cross section are coupled to each other via the rigid phosphate groups (see Figure 6a). Thus, the observed O^2–^ ordering and the unique site decoration pattern necessitate each other resulting in the observed apatite superstructure combined with a lowered space group symmetry in Pb_10-x_Cu_*x*_(PO_4_)_6_O with *x* < 0.5. To the best of our knowledge, a structure like the one described here for Pb_10-x_Cu_*x*_(PO_4_)_6_O with *x* < 0.5 has never been described before.

Most modulated apatite structure are hydroxide containing apatites with ordered orientation of the (OH)^−^ in the hexagonal channels, which leads to a doubled *b* lattice parameter and a reduction in symmetry to a monoclinic cell.^10,20^ Out of the modulated apatites that retain hexagonal symmetry, the Ba_5_(VO_3_S)_3_F-type and Ba_10_(VO_3_S)_6_S-type structures form commensurate 2 × 2 × 1 superstructures because of vacancy ordering in the *ab*-plane while Cd_5_(PO_4_)_3_Br and Cd_5_(VO_4_)_3_I apatites exhibit incommensurate modulations in the *ab*-plane.^29−33^ In contrast, the ordering in Pb_10-x_Cu_*x*_(PO_4_)_6_O with *x* < 0.5 reported here occurs along the *c* direction. Distinctly different ordering patterns along the *c* direction have been observed in the La_3_Nd_11_(SiO_4_)_9_O_3_-type and Ca_15_(PO_4_)_9_IO-type structures.^29,30^ The former exhibits ordering of the La^3+^ and Nd^3+^ cations on the A1 sites with the A2 sites fully occupied by Nd^3+^, while the latter shows anion-vacancy ordering in the hexagonal channels with the A1 and A2 sites being fully occupied by Ca^2+^. The commensurate modulations in both structures result in 1 × 1 × 3 superstructures in space group *P*6_3_/*m*. In contrast, the 1 × 1 × 2 superstructure in space group *P*6̅ reported here for Pb_10-x_Cu_*x*_(PO_4_)_6_O with *x* < 0.5 arises from the combination of Cu^2+^ cations occupying only one of the two stacks of trigonal metaprisms (sites Pb1–2 and Pb1–4) with the anion-vacancy ordering in the hexagonal channels.

Moreover, Pb_10-x_Cu_*x*_(PO_4_)_6_O with *x* < 0.5 is also distinctly different from Pb_10–x_Cu_*x*_(PO_4_)_6_O with 0.9 < *x* < 1 as reported Puphal et al. as the latter contains significantly more copper, does not show the same site decoration pattern and exhibits no vacancy ordering.^4^ Critically, Pb_10–x_Cu_*x*_(PO_4_)_6_O with 0.9 < *x* < 1 crystallizes in the archetypical apatite structure in space group *P*6_3_/*m* with Cu^2+^ substituting Pb^2+^ on both the A1- and the A2-site (10(6) % and 14(6)%, respectively) resulting in the refined single crystal composition Pb_8.8(3)_Cu_1.2(3)_(PO_4_)_6_O, whereas only two out of the four A1-sites are occupied by Cu^2+^ in Pb_10-x_Cu_*x*_(PO_4_)_6_O with *x* < 0.5 reported here. The remaining A1 and all A2-sites of Pb_10-x_Cu_*x*_(PO_4_)_6_O with *x* < 0.5 are exclusively occupied by Pb^2+^ yielding the composition Pb_9.842(8)_Cu_0.158(8)_(PO_4_)_6_O. This unique site decoration pattern also drives the symmetry reduction to space group *P*6̅ (no. 174). Another difference lies in the oxide anions in the hexagonal channels of Pb_10–x_Cu_*x*_(PO_4_)_6_O with 0.9 < *x* < 1, which fully occupy the 2*b* site located at (0,0,0). In contrast, the oxide anions in the hexagonal channels of Pb_10–x_Cu_*x*_(PO_4_)_6_O with *x* < 0.5 are all partially occupied and displaced from (0,0,0) as highlighted in Figure 4. This commensurate modulation along the channel axis gives rise to the 1 × 1 × 2 superstructure in Pb_10–x_Cu_*x*_(PO_4_)_6_O with *x* < 0.5.

To verify the composition obtained from single crystal diffraction, the Pb:Cu ratio of several single crystals was probed by EDX (see Figure S2a). The Pb:Cu ratio varies widely between different single crystals, showing that Cu is not homogeneously distributed throughout the sample. The average Pb:Cu ratio from EDX is Pb7(2):Cu3(2). This is in reasonable agreement with the compositions obtained from the Rietveld refinement of the powder diffraction data. Looking at the data in detail reveals that the data are not evenly distributed but are divided into three different clusters (see Figure S2b) with the average metal ratios corresponding to Pb_9.8(2)_Cu_0.2(2)_(PO_4_)_6_O, Pb_7.7(2)_Cu_2.3(2)_(PO_4_)_6_O and Pb_3.9(3)_Cu_6.1(3)_(PO_4_)_6_O reflecting the compositions refined for different single crystals within the sample (see Figure 5b).

Plotting the average occupancies for equivalent sites in Figure 5b clearly shows the strong site preference of Cu^2+^ for the trigonal prismatic A1 sites for both structures. Combined with the coupling of the modulated channel cross section to the variations in the twist angles, this accounts for why the superstructure in the solid solution Pb_10-x_Cu_*x*_(PO_4_)_6_O is only observed for small *x* (Figure 5b). For small x, Cu occupies the Pb1–2 and Pb1–4 sites (2*i*) exclusively due to the larger flexibility of this stack of metaprisms as indicated by the corresponding twist angles being both the smallest and the largest (see coordination of Pb1–2 and Pb1–4 in the Supporting Information, Figure S4) resulting in the unique site decoration pattern of Pb_10-x_Cu_*x*_(PO_4_)_6_O with *x* < 0.5. The twist angle ϕ = 24.5(7)° of the Pb1–2 metaprism for *x* < 0.5 however is close to the upper limit of the commonly observed range for ϕ.^39^ Consequently, it is reasonable to suggest that the twist angle ϕ limits how much of the smaller Cu can be incorporated into this site in the superstructure. Presumably, the difference in twist angle becomes too large if more Cu is incorporated on this site if the lower symmetry structure were retained. Thus, Cu is eventually forced onto the other trigonal prismatic sites (Pb1–1 and Pb1–3) in the other stack of prisms (see Figure 6b) to avoid an overlarge strain at the restricted set of trigonal metaprismatic sites Pb1–2 and Pb1–4 that might arise if more Cu were solely incorporated on those sites beyond x = 0.5. Instead, Cu is then distributed over a larger number of the available metal sites in the structure in order to reduce the strain. As the four A1 sites (Pb1–1, Pb1–2, Pb1–3 and Pb1–4) become equivalent in the site Pb1 of Pb_10-x_Cu_*x*_(PO_4_)_6_O with *x* > 0.5, the space group symmetry changes from *P*6̅ to *P*6_3_/*m.* Since the twist of trigonal metaprisms is coupled to the channel cross section by the phosphate groups coordinating to the A2 cations, the channel cross section becomes constant, when the trigonal metaprisms are all identical, which subsequently alters the O^2–^ ordering in Pb_10-x_Cu_*x*_(PO_4_)_6_O with *x* > 0.5. Thus, exceeding the threshold for Cu on the Pb1–2 and Pb1–4 sites that is set by the twist angle then forces Cu to occupy all the metaprisms, which changes the channel cross section from a modulated to a constant channel size and thus suppresses the oxygen anion ordering found in Pb_10-x_Cu_*x*_(PO_4_)_6_O with *x* < 0.5 when *x* > 0.5. The outlined mechanism explains the observed cation- and anion-ordering through coupling via the twist angle in Pb_10-x_Cu_*x*_(PO_4_)_6_O with *x* < 0.5 and the transition to the apatite archetype structure in Pb_10-x_Cu_*x*_(PO_4_)_6_O with *x* > 0.5.

### Magnetization

**Figure 7:**
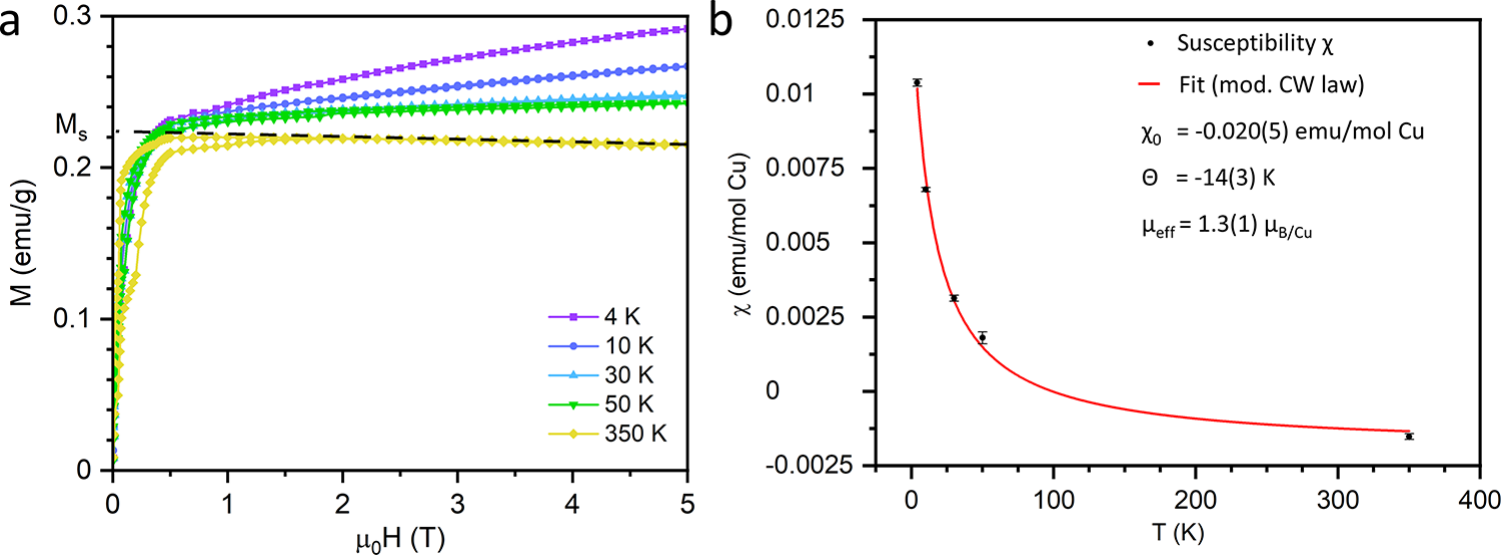
Field-dependent magnetization at different temperatures (a). The dashed line highlights the decreasing magnetization at 350 K with the intercept corresponding to M_s_. The temperature-dependent susceptibility (b) extracted from the Honda-Owen plot is fitted to a modified Curie–Weiss law (red line).

Field-dependent magnetization M(H) and temperature-dependent susceptibility of the powdered sample containing both apatite structures as well as Pb_3_(PO_4_)_2_ are presented in Figure 7. The hysteresis loops show a ferromagnetic and a diamagnetic component at high temperatures. Plotting the magnetization M for H > 1T, the saturated magnetization of the ferromagnetic component M_S_ and the susceptibility χ(T) can be obtained using the Honda-Owen method (see Experimental section for details).^41^ The saturated magnetization M_S_ is equivalent to ca. 0.3 μmol or roughly 17 μg Fe, which is plausible for a small ferromagnetic contamination. Thus, M_S_ is not associated with Pb_10-x_Cu_*x*_(PO_4_)_6_O but a ferromagnetic impurity that is below the detection limit of the PXRD. The susceptibility χ(T) was fitted using a modified Curie–Weiss law of the form χ(T) = χ_0_ + C/(T-Θ).^42^

Furthermore, a diamagnetic component causes M to decrease linearly at large magnetic fields visible in the hysteresis loop at 350 K (Figure 7a). Fitting the susceptibility χ(T) yields χ_0_ = −0.020(5) emu/mol Cu. This is consistent with the expected Pascal’s constant of −0.012 emu/mol Cu for Pb_9.5_Cu_0.5_(PO_4_)_6_O.^43^ The paramagnetic tail observed at low temperatures in the susceptibility χ(T) is attributed to the Cu^2+^ cation (3d^9^). The effective magnetic moment μ_eff_ = 1.3(1) μ_B_/mol Cu is 75% of the theoretical moment of 1.73 μ_B_ /Cu^2+^ cation.^18^

Importantly, the sample’s magnetic response shows no sign of superconductivity since the diamagnetic component can simply be attributed to the intrinsic diamagnetism of the constituent atoms. Superconductivity in Pb_2_Sr_2_(Y,Ca)Cu_3_O_8+δ_ and other cuprates is associated with strongly interacting copper cations. The crystal structures comprise of 2D layers of mixed-valent [CuO] motifs with the amount of oxide vacancy concentration playing a pivotal role for the superconducting properties.^8^ In contrast, the metal cations in apatites are arranged in 1D chains and the substitution of Pb by Cu does not exceed 22.8(3) and 7.9(6) % for Pb_10-x_Cu_*x*_(PO_4_)_6_O with *x* > 0.5 and for Pb_10-x_Cu_*x*_(PO_4_)_6_O with *x* < 0.5 respectively. Therefore, the interactions between the copper cations in the materials studied here are insufficient to generate the necessary delocalized behavior required for superconductivity. This is consistent with the localized electron Curie–Weiss magnetism observed once adventitious ferromagnetism is accounted for.

## Conclusions

We have synthesized single crystals of Pb_10-x_Cu_*x*_(PO_4_)_6_O in a wide compositional range. For *x* < 0.5, we observe a unique structure type in the apatite family. It is the first 1 × 1 × 2 superstructure of the archetypical hexagonal apatite structure with the space group symmetry lowered from *P*6_3_/*m* to *P*6̅. The Cu^2+^ cations partially occupy only two of the available seven metal cation sites. The accompanying subtle distortions accommodate a unique combination of O^2–^ ordering pattern and cation site order. The structure of the solid solution Pb_10-x_Cu_*x*_(PO_4_)_6_O changes to the apatite archetype structure in *P*6_3_/*m* for *x* > 0.5. This is attributed to Cu being forced to partially occupy all of the available metal cation sites in order to avoid excessive strain through twist distortion of a restricted subset of the trigonal prism sites. The study exemplifies the synergic distortions that can arise from coupling of the core apatite structural units with the rigid tetrahedral [PO_4_]^3–^ anions forming a linkage between the cations in trigonal metaprismatic coordination and the anions occupying the 1D channels. In context of recent reports of room-temperature superconductivity in LK-99 containing lead apatite solid solutions, this study does not observe superconducting behavior in the sample, highlighting the absence of infinite [CuO] motifs in Pb_10-x_Cu_*x*_(PO_4_)_6_O with small x as a key structural difference to well-known cuprate superconductors.

## Experimental Section

### Synthesis

Targeting the composition Pb_9.5_Cu_0.5_(PO_4_)_6_O, lead oxide (PbO, Alfa Aesar, > 99%), cupric oxide (CuO, Sigma-Aldrich, > 99%) and ammonium dihydrogenphosphate (NH_4_H_2_PO_4_, Alfa Aesar, > 98%) powders were mixed in the corresponding molar ratio, pressed into a pellet and placed in an alumina crucible. The sample was heated in air using an electric muffle furnace. First, the sample was heated to 300 °C for 4 h to decompose NH_4_H_2_PO_4_ into HPO_3_ and H_2_O before the temperature was raised to 925 °C for 48 h. The furnace then was cooled back to room temperature at a rate of 100 °C/h. The samples were extracted from the crucible, which produced crystalline fragments suitable for single crystal X-ray diffraction. The majority of each sample was ground into powder for further measurements.

*Warning: lead compounds are toxic, and they cannot be released into the environment. It is important to handle all samples in well-ventilated fume hoods and to wear proper personal protective equipment. All contaminated waste must be appropriately disposed of.*

### Powder Diffraction

Powder diffraction (PXRD) was carried out at Diamond Light Source, U.K., on the high-resolution beamline I11 using synchrotron radiation with a wavelength of λ = 0.82385 Å.^44^ The finely ground powder was dusted onto the outer surface of 0.2 mm ID borosilicate glass capillary, which was then inserted into a 1 mm ID borosilicate glass capillaries. This procedure provides a very thin layer of powder minimizing the absorption due to lead. The diffraction pattern was recorded in the transmission mode [2° < 2θ < 92.4°] using a position sensitive detector (PSD) at room temperature. A Rietveld refinement was carried out using FullProf Suite.^45^

### Single Crystal Diffraction

Initially, single crystals were manually extracted from the sample and screened in-house on a Rigaku single crystal diffractometer with AFC12K goniometer, a molybdenum rotating anode microfocus X-ray source and Hypix-6000HE Hybrid detector.

Suitable single crystals both with and without superstructure reflections were sent to Diamond Light Source, U.K., for single crystal diffraction experiments with monochromatic synchrotron radiation (λ = 0.6889 Å). The diffraction data were collected at 100 K at beamline I19, which is equipped with a Pilatus 2 M detector.^44,46,47^ Data integration and absorption correction were performed using Xia27 and Dials programs.^48−50^ The structures were solved by the intrinsic phasing method as implemented in SHELXT^51^ and refined with SHELXL.^52^ Space group symmetries were confirmed using the ADDSYM procedure in Platon.^53^ No additional symmetry operations were detected. The recommended origin shift (1/3, 2/3, 0) for Pb_10-x_Cu_*x*_(PO_4_)_6_O with *x* < 0.5 was not implemented to keep the unit cell settings comparable to the subcell.

Pb_10-x_Cu_*x*_(PO_4_)_6_O with *x* > 0.5: All atoms are refined with anisotropic displacement parameters except for two partially occupied O sites. The sum of occupancies of the partially occupied O sites has been constrained to one O^2–^ per formula unit to maintain charge neutrality. The metal sites have been tested for occupational disorder and the occupancies of both sites were refined independently constraining the total occupancy of either site to 100%. The occupancies of the split Pb2 site are refined using the SUMP instruction. The anisotropic displacement parameters of Cu and Pb are constrained to be identical for each cation pair. The occupancies of the O and P sites forming the tetrahedral phosphate groups are not refined.

Pb_10-x_Cu_*x*_(PO_4_)_6_O with *x* < 0.5: The metal cations were refined using anisotropic displacement parameters, whereas the light elements P and O are refined with isotropic displacement parameters. The occupancies of all metal sites were refined freely to test for mixed occupancies before fixing the occupancies to 100% if no significant deviation from 100% was observed. After confirming that the occupancies of the Pb1–2 and Pb1–4 sites are the identical within the margin of error by refining their occupancies independently, the occupancies of both sites have been refined using a single variable. The occupancies of the O and P sites forming the tetrahedral phosphate groups are not refined. The split Pb site is modeled with three partially occupied sites using a single variable. The sum of the occupancies was constrained to 100% during the structural refinement. The occupancies of the three sites O^2–^ sites in the 1D channels are refined employing a SUMP instruction to constrain their sum to maintain charge balance.

### Energy-Dispersive X-ray Spectroscopy

After collecting single crystal X-ray diffraction data, crystals of Pb_10-x_Cu_*x*_(PO_4_)_6_O were rinsed with acetone and coated with carbon as preparation for energy dispersive X-ray spectroscopy (EDX). EDX spectra were collected with a Hitachi S4800 scanning electron microscope using the AZtecOne software, at a 15.0 mm distance and a beam voltage of 20.0 kV to obtain the elemental composition.

### Magnetization Measurements

The temperature dependent magnetization of the sample was measured using a Magnetic Property Measurement System (MPMS) by Quantum Design in the range from 2 to 350 K. Hysteresis loops in the range of −5 to 5 T were recorded at 4 K, 10 K, 30 K, 50 and 300 K. Subsequently, the saturation magnetization M_s_ was extracted at H = 1 T, 2 T, 3 T, 4 and 5 T and the susceptibility χ(T) calculated using Honda-Owen method from the slope for μ_0_H > 2.5 T.^41^ The susceptibility χ(T) was fitted to a modified Curie–Weiss law.^42^

## Data Availability

The data that support the findings of this study are openly available in the University of Liverpool Research Data Catalogue at https://datacat.liverpool.ac.uk/id/eprint/2846. The crystal structures for Pb_9.842(8)_Cu_0.158(8)_(PO_4_)_6_O and Pb_8.68(3)_Cu_1.32(2)_(PO_4_)_6_ O are deposited with CSD accession codes 2394906 and 2394905, respectively.

## Supporting Information Available

Crystal structures, PXRD data, electron density maps, and crystallographic details (PDF)
Crystallographic data of Pb_8.68(3)_Cu_1.32(2)_(PO_4_)_6O_ in *P*6_3_/*m* (CIF)
Crystallographic data of Pb_9.842(8)_Cu_0.158(8)_(PO_4_)_6O_ in *P*6̅ (CIF)

## Supplementary Material

cm4c03130_si_001.pdf

cm4c03130_si_002.cif

cm4c03130_si_003.cif

## Footnotes

‡ Throughout the manuscript, we will refer to the site as “Pb2–3” if the structural discussion does not require the distinction between the split sites.
