# Supplementary material for: Superstructure Formation through Coupled Anion and Cation Ordering in Cu-Substituted Lead Oxyapatites
Source: Chem Mater. 2025 Apr 23;37(9):3088–99. doi: 10.1021/acs.chemmater.4c03130 (PMC12079795; doi:10.1021/acs.chemmater.4c03130)
Supplement: Supplementary file 1 — cm4c03130_si_001.pdf [file cm4c03130_si_001.pdf]

Superstructure Formation through Coupled Anion and Cation Ordering in Cu-substituted Lead Oxyapatites – Supporting Information

Jan P. Scheifers,[a,b] Adam J. D. Richardson,[b] Hai Lin,[a,b] Hongjun Niu,[b] Luke M. Daniels,[b] Michael W. Gaultois,[a,b] Jonathan Alaria,[c] Craig M. Robertson,[b] John B. Claridge,[b] and Matthew J. Rosseinsky[a,b]*

[a] Leverhulme Research Centre for Functional Materials Design, University of Liverpool, Materials Innovation Factory, Liverpool L69 7ZD, United Kingdom.

[b] Department of Chemistry, University of Liverpool, Crown Street, L69 7ZD, United Kingdom.

[c] Department of Physics, University of Liverpool, The Oliver Lodge Laboratory, Oxford Street, Liverpool L69 7ZE, United Kingdom.

Figure S1: powder synchrotron diffraction pattern and simulated powder diffraction patterns (inset) of archetypical $Pb_{8.68(3)}Cu_{1.32(2)}(PO_4)_6O$ and $Pb_{9.842(8)}Cu_{0.158(8)}(PO_4)_6O$ with superstructure

Figure S2: a) SEM image and EDX spectrum, b) distribution of normalized metal ratios from EDX data

Figure S3: electron density maps of the (110) planes

Figure S4: trigonal metaprismatic coordination environments

Figure S5: pentagonal pyramidal coordination coordination environments

Table S1: atomic coordinates, occupancies and displacement parameters obtained from single crystal diffraction

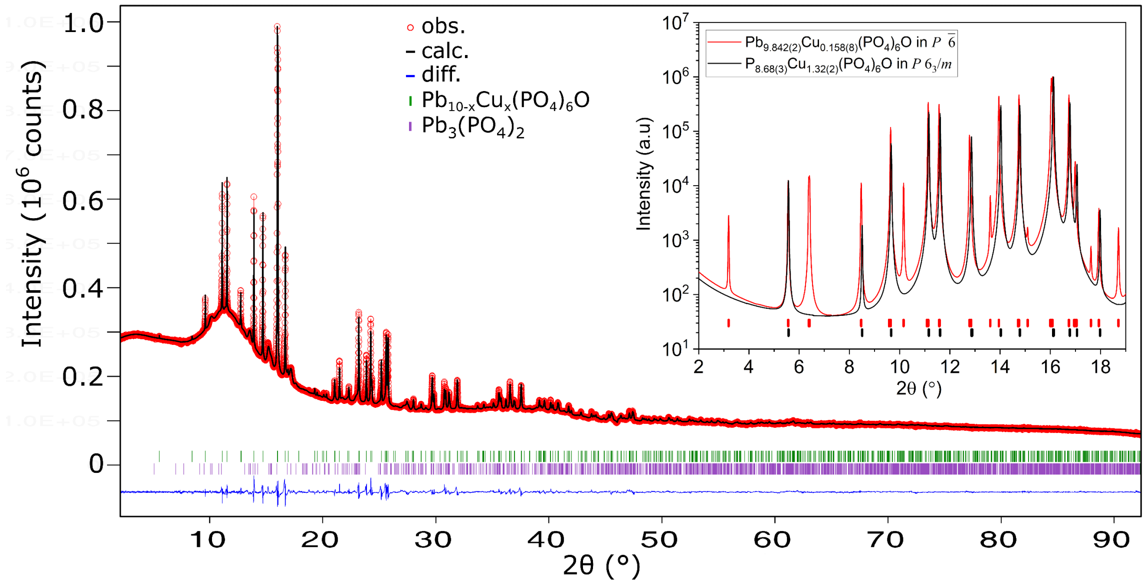

Figure S1: refined synchrotron PXRD data (red circles) of a sample containing 79(1) wt.% of the oxyapatite $Pb_{8.66(5)}Cu_{1.33(5)}(PO_4)_6O$ and 20.7(4) wt.% $Pb_3(PO_4)_2$. The fit to the data is shown as a black line and the difference is plotted in blue. The green tick marks correspond to the reflection positions of $Pb_{8.66(5)}Cu_{1.33(5)}(PO_4)_6O$ in $P6_3/m$, purple tick marks to $Pb_3(PO_4)_2$., respectively. Inset: logarithmic scale comparison of the simulated PXRD patterns of archetypical $Pb_{8.68(3)}Cu_{1.32(2)}(PO_4)_6O$ and $Pb_{9.842(8)}Cu_{0.158(8)}(PO_4)_6O$ with superstructure simulated based on the respective single crystal structures. The largest superstructure reflections are of the same scale as the smallest reflections of the archetype and thus undetectable in the experimental data.

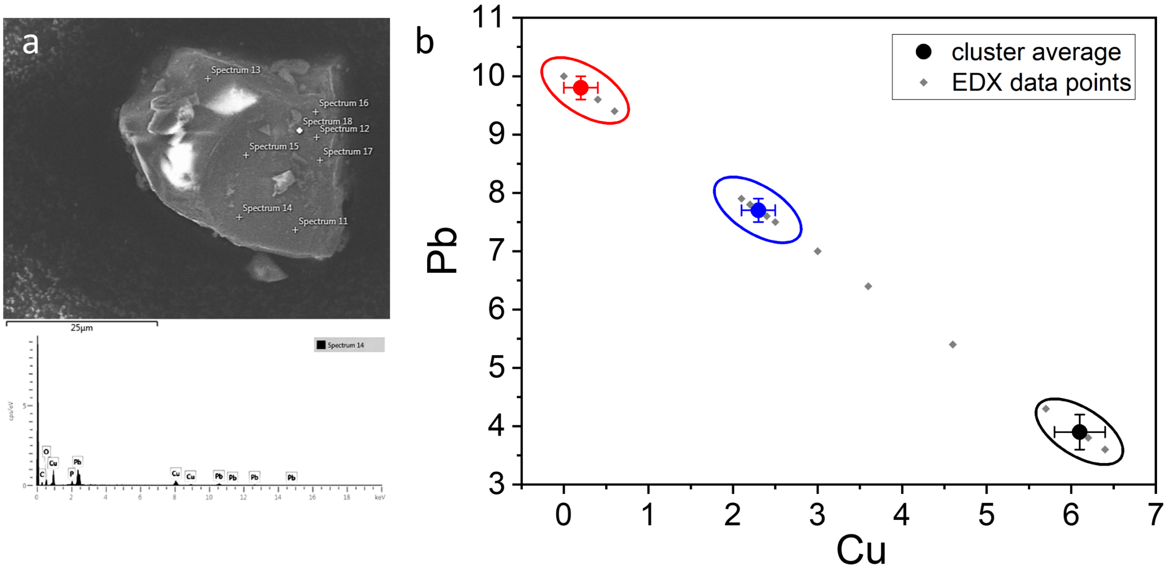

Figure S2: Panel (a) shows SEM image of $Pb_{10\text{-}x}Cu_x(PO_4)_6O$ crystal (top) and representative EDX spectrum (below). Panel (a) shows the amount of Pb and Cu in $Pb_{10\text{-}x}Cu_x(PO_4)_6O$ crystals as determined by EDX (grey) as well as three distinct clusters with their corresponding averages (red, blue and black).

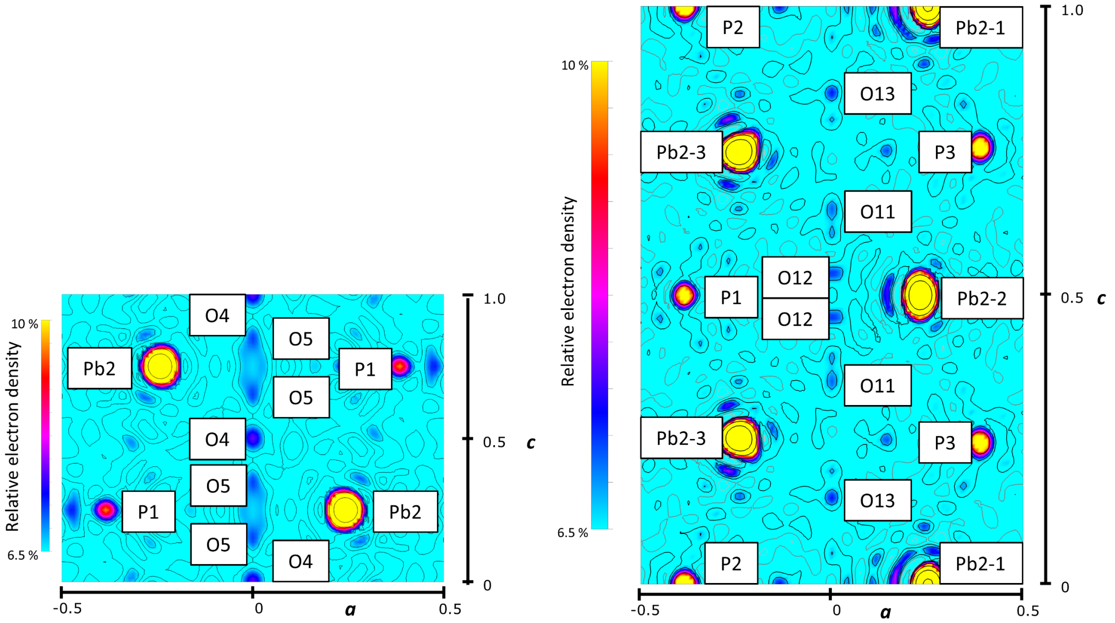

Figure S3: electron density maps of $Pb_{8.68(3)}Cu_{1.32(2)}(PO_4)_6O$ (left) and $Pb_{9.842(8)}Cu_{0.158(8)}(PO_4)_6O$ (right) obtained from single crystal X-ray diffraction data. The electron density maps show the respective (110) unit cell cross sections centred around the 1D channels of the two apatite structures. Between the yellow maxima of the heavy Pb^{2+} cations dark blue maxima are clearly visible along the c-direction indicating the presence of the lighter oxide anions. Some of the O^{2-} disorder visible in $Pb_{8.68(3)}Cu_{1.32(2)}(PO_4)_6O$ is resolved in $Pb_{9.842(8)}Cu_{0.158(8)}(PO_4)_6O$ by creating a 1×1×2 superstructure. The electron density around the Pb2-3-site in $Pb_{9.842(8)}Cu_{0.158(8)}(PO_4)_6O$ is not ellipsoidal indicating site splitting.

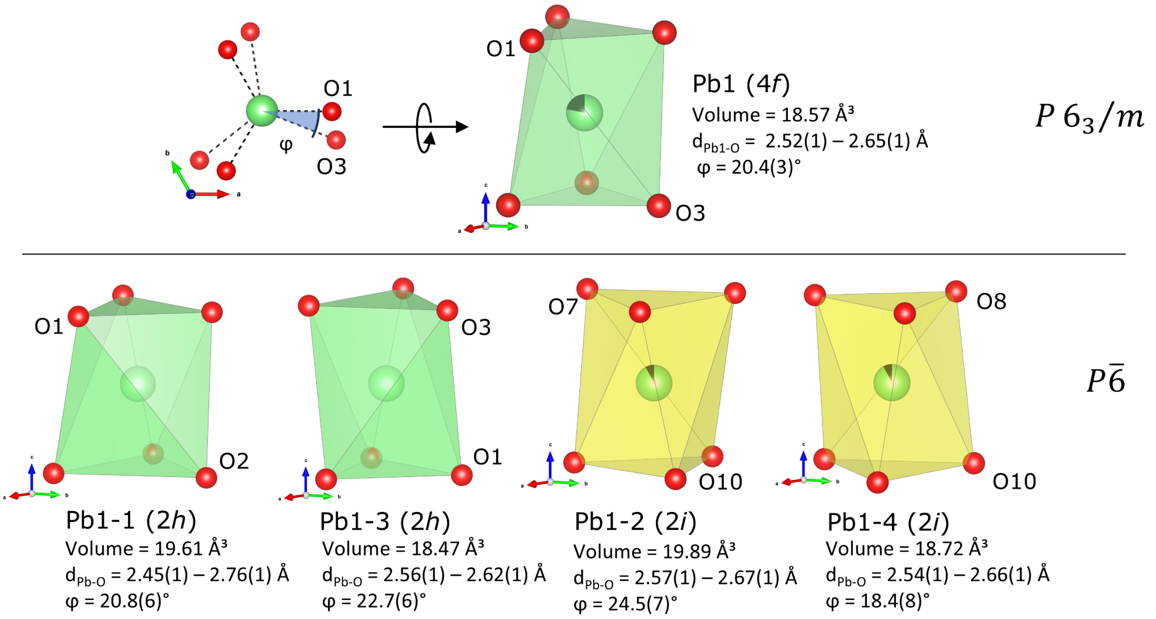

Figure S4: trigonal metaprismatic coordination(s) of the A1 cations in $Pb_{10-x}Cu_x(PO_4)_6O$ with x > 0.5 (top) and $Pb_{10-x}Cu_x(PO_4)_6O$ with x < 0.5 (bottom) with twist angle ϕ.

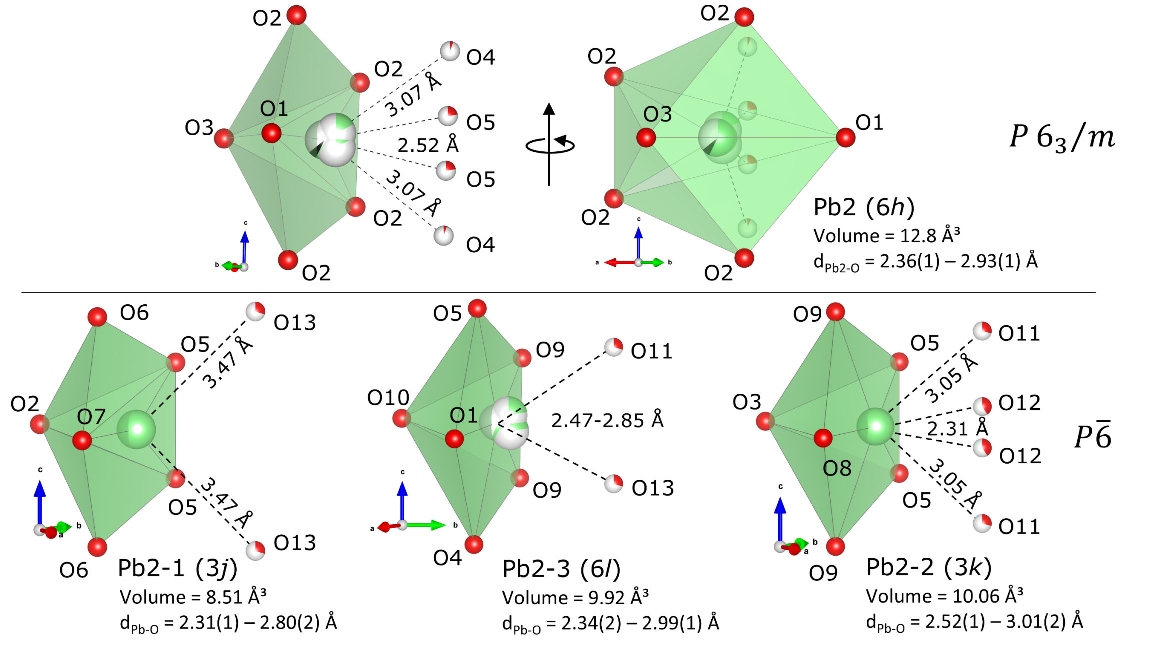

Figure S5: distorted pentagonal pyramidal coordination(s) of the A2 cations in $Pb_{10-x}Cu_x(PO_4)_6O$ with x > 0.5 (top, two views) and $Pb_{10-x}Cu_x(PO_4)_6O$ with x < 0.5 (bottom) with the distances to the partially occupied O^{2-} sites in the channels.

Table S1: Wyckoff sites, fractional coordinates, occupancies and equivalent displacement parameters U_{eq} for all atoms in $Pb_{9.842(8)}Cu_{0.158(8)}(PO_4)_6O$ *(space group* $P\bar{6}$*) and* $Pb_{8.68(3)}Cu_{1.32(2)}(PO_4)_6O$ *(space group* $P6_3/m$*) as obtained from synchrotron single-crystal diffraction.*

site	Wyckoff site	*x*	*y*	*z*	Occupancy [%]	U_{eq} [Å^2]
			$Pb_{9.842(8)}Cu_{0.158(8)}(PO_4)_6O$ in $P\bar{6}$			
Pb1-1	*2h*	⅓	⅔	0.1289(1)	100 Pb	0.006(1)
Pb1-3	*2h*	⅓	⅔	0.3766(1)	100 Pb	0.009(1)
Pb2-1	*3j*	0.0006(1)	0.2511(1)	0	100 Pb	0.008(1)
Pb2-2	*3k*	0.2330(1)	0.0035(1)	½	100 Pb	0.018(1)
Pb2-3A	*6l*	0.2527(4)	0.2524(4)	0.2523(1)	56(1) Pb	0.005(1)
Pb2-3B	*6l*	0.2232(4)	0.2266(5)	0.2523(1)	22.2(5) Pb	0.005(1)
Pb2-3C	*6l*	0.2273(5)	0.2298(5)	0.2630(3)	22.2(5) Pb	0.005(1)
Pb1-2	*2i*	⅔	⅓	0.1241(1)	92.1(6) Pb<br>7.9(6) Cu	0.007(1)<br>0.007(1)
Pb1-4	*2i*	⅔	⅓	0.3779(1)	92.1(6) Pb<br>7.9(6) Cu	0.009(1)<br>0.009(1)
P1	*3k*	0.3721(6)	0.4024(5)	½	100 P	0.003(1)
P2	*3j*	0.3750(6)	0.4002(5)	0	100 P	0.003(1)
P3	*6l*	0.4019(5)	0.0263(6)	0.2459(2)	100 P	0.007(1)
O1	*6l*	0.1533(12)	0.4891(13)	0.2450(4)	100 O	0.011(2)
O2	*3j*	0.1006(12)	0.5202(13)	0	100 O	0.005(2)
O3	*3k*	0.1233(12)	0.5205(13)	½	100 O	0.007(2)
O4	*6l*	0.2590(9)	0.3375(9)	0.0799(5)	100 O	0.015(2)
O5	*6l*	0.2559(9)	0.3274(8)	0.4200(5)	100 O	0.013(2)
O6	*6l*	0.3637(7)	0.0877(7)	0.1585(4)	100 O	0.001(1)
O7	*3j*	0.4766(13)	0.3203(12)	0	100 O	0.007(2)
O8	*3k*	0.4900(15)	0.3430(15)	½	100 O	0.011(2)
O9	*6l*	0.3539(8)	0.0853(8)	0.3318(5)	100 O	0.011(2)
O10	*6l*	0.5869(15)	0.1139(15)	0.2514(4)	100 O	0.023(3)
O11	*2g*	0	0	0.362(3)	29.4(4) O	0.011(4)
O12	*2g*	0	0	0.470(2)	40.8(4) O	0.011(4)
O13	*2g*	0	0	0.165(3)	29.8(4) O	0.011(4)

$Pb_{8.68(3)}Cu_{1.32(2)}(PO_4)_6O$ in $P6_3/m$						
Pb1	*4f*	⅓	⅔	0.4962(1)	77.2(3) Pb<br>22.8(3) Cu	0.009(1)<br>0.009(1)
Pb2	*6h*	0.0010(2)	0.2516(1)	¾	57.4(3) Pb<br>6.8(3) Cu	0.008(1)<br>0.008(1)
Pb2S	*12i*	0.2263(3)	0.2294(2)	0.2309(3)	17.9(1) Pb	0.008(1)
P1	*6h*	0.3759(1)	0.4026(1)	¾	100 P	0.008(1)
O1	*6h*	0.4886(4)	0.3351(5)	¾	100 O	0.018(1)
O2	*12i*	0.2677(3)	0.3486(4)	0.5815(3)	100 O	0.020(1)
O3	*6h*	0.4712(5)	0.5856(4)	¾	100 O	0.025(1)
O4	*2b*	0	0	½	5(2) O	0.025(5)
O5	*4e*	0	0	0.176(3)	22.5(9) O	0.025(5)

Reference databases last accessed on 02/05/2024:

- Inorganic Crystal Structure Database (ICSD, release 2024.1)
- Pearson's Crystal Data - Crystal Structure Database for Inorganic Compounds, release 2023/24
